# Radioprotection Performance Evaluation of 3D-Printed and Conventional Heat-Cured Dental Resins for Radiotherapy Prostheses

**DOI:** 10.3390/jfb15100282

**Published:** 2024-09-25

**Authors:** Jiangyu Wang, Mai Murase, Yuka I. Sumita, Ryoichi Notake, Masako Akiyama, Ryoichi Yoshimura, Noriyuki Wakabayashi

**Affiliations:** 1 Department of Advanced Prosthodontics, Graduate School, Medical and Dental Sciences, Tokyo Medical and Dental University, Tokyo 1138510, Japan; jiang.mfp@tmd.ac.jp (J.W.); wakabayashi.rpro@tmd.ac.jp (N.W.); 2 Department of Partial and Complete Denture, The Nippon Dental University School of Life Dentistry, Tokyo 1028159, Japan; sumita@tky.ndu.ac.jp; 3 Radiology Center, Graduate School, Medical and Dental Sciences, Institute of Research, Tokyo Medical and Dental University, Tokyo 1138510, Japan; ntkcrad@tmd.ac.jp (R.N.); ysmrmrad@tmd.ac.jp (R.Y.); 4 Young Investigator Support Center, Graduate School, Medical and Dental Sciences, Tokyo Medical and Dental University, Tokyo 1138510, Japan; m-akiyama.cell@tmd.ac.jp; 5 Department of Radiation Therapeutics and Oncology, Graduate School, Medical and Dental Sciences, Tokyo Medical and Dental University, Tokyo 1138510, Japan

**Keywords:** 3D printing, dental resin, dentistry, radiation attenuation, radiotherapy prostheses

## Abstract

3D printing is increasingly used in dentistry, with biocompatible resins playing a key role. This study compared the radioprotective properties of a commonly used 3D-printed resin (Formlabs surgical guide resin) with traditional heat-cured resin and examined the relationship between material thickness and radiation attenuation. The specimens consisted of 3D-printed and heat-cured resin specimens, each measuring 45 × 45 mm^2^, with five different thicknesses (6, 8, 10, 12, and 14 mm), totaling 100 samples. Both types of resin specimens underwent testing with 150 MU external beam radiation therapy (EBRT) and 400 cGy brachytherapy. Radiation experiments indicated that under EBRT conditions, there were no significant differences in radiation attenuation between the 3D-printed and heat-cured resins across all thickness groups. In brachytherapy, the attenuation of the 3D-printed resin was significantly lower than the heat-cured resin in the 6 mm and 8 mm groups. Specifically, attenuation rates were 48.0 ± 0.7 (3D-printed) vs. 45.2 ± 1.9 (heat-cured) in the 6 mm group, and 39.6 ± 1.3 vs. 37.5 ± 1.1 in the 8 mm group. Both resins showed significant positive linear correlations between thickness and attenuation (*p* < 0.001) within 6–14 mm. Thus, 3D-printed resin shows promising radioprotective properties and is a viable alternative to traditional heat-cured resin.

## 1. Introduction

**Figure 1:**
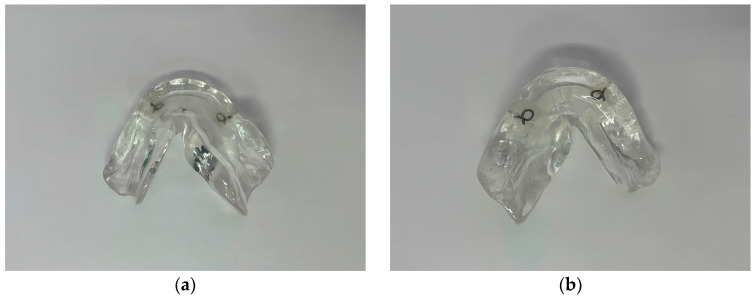
Radiotherapy prosthesis made from heat-cured resin. (**a**) Intaglio surface; (**b**) polished surface.

Three-dimensional (3D) printing has become increasingly important in dentistry for fabricating customized dental elements, owing to its superior design flexibility. It facilitates the creation of complex geometric shapes with high manufacturing efficiency, enabling faster prototyping and small-batch production [1,2]. The evolution of digital dentistry has led to the development and use of novel materials, including biocompatible resins. One such material, often used in 3D printing, is surgical guide resin (Formlabs Inc., Somerville, MA, USA). According to the manufacturer, this biocompatible material is suitable for short-term use, offering strength, rigidity, transparency, and durability against common disinfectants and sterilization agents [3]. Although initially introduced for producing surgical guides used in implantology and craniofacial surgery [4,5], this material has recently found applications in radiology, particularly for manufacturing radiation protection devices for patients with head and neck cancer [6,7]. Radiotherapy is a key treatment for head and neck cancer patients [8], helping to preserve the form of affected tissues and thereby avoid the significant tissue loss and psychological impact that can result from surgical tumor removal in this area [9,10,11]. Radiotherapy encompasses external beam radiation therapy (EBRT) and brachytherapy, which includes low-dose-rate and high-dose-rate brachytherapy (HDR). Linear accelerators are widely used in EBRT to generate high-energy X-rays from a distance and use these photon beams to precisely target cancer cells, thereby controlling, reducing, or eliminating tumors [12]. In HDR, commonly used Ir-192 radiation sources emit γ-rays, another form of photon beam, and are strategically placed within or near tumor tissues to precisely deliver high-dose radiation [13]. However, radiotherapy is also associated with complications such as radiation-induced osteonecrosis, mucositis, xerostomia, and radiation caries [14]. To minimize these side effects, the fabrication of radiotherapy prostheses is recommended and the need for radiotherapy prostheses is well documented. These devices are designed to shield tissues surrounding the target area during radiation therapy, helping to minimize the adverse effects of treatment [15,16,17,18,19]. Although heat-cured resin has traditionally been used to manufacture radiotherapy prostheses (Figure 1) [19,20,21,22], 3D printing is increasingly preferred for its higher accuracy, precision, and shorter processing time [2].

Most studies investigating novel biocompatible 3D-printed resins have focused on evaluating their physical and mechanical properties [23,24]. However, despite some papers discussing the use of 3D printing in radiotherapy for the head and neck area [6,7], there have been few papers specifically addressing the crucial parameters, such as radiation attenuation, that define the radioprotective properties of 3D-printed resin materials. It remains unclear whether 3D-printed resin can effectively replace conventional heat-cured PMMA in terms of radiation attenuation. Radiation attenuation refers to the reduction in the strength or intensity of radiation as it passes through a material. The commonly accepted definition of attenuation is the ratio of the transmitted beam intensity *(I*) to the initial beam intensity (*I*_0_). It is well-established that for monoenergetic radiation, attenuation follows an exponential relationship, which is influenced by both the material’s linear attenuation coefficient (*μ*) and its thickness (∆*x*). As radiation passes through a material, its intensity decreases exponentially, depending on the properties of the material and the thickness it traverses [25]. Specifically, attenuation is proportional to both the density and the atomic number of the elements within the material. Using homogeneous materials ensures a predictable and consistent level of radiation attenuation, which is crucial for effective radiation shielding and protection [26,27,28]. Also, the radiation source [27,28] and the thickness of the material [29] are critical factors that affect the radiation attenuation properties of materials. Therefore, in this study, the radiation attenuation of 3D-printed resin at five different thicknesses was validated and compared with that of heat-cured resin under both EBRT and HDR radiation sources. This helps dentists and radiologists to select the appropriate material and determine the thickness radiation prostheses based on individual patient needs.

The aim of this study is to explore the feasibility of using 3D-printed resin to produce radiotherapy prostheses and establish a reliable relationship between material thickness and radiation attenuation within clinically relevant thickness ranges.

In this study, with the understanding that the relationship between attenuation and thickness is exponential, the following hypotheses were tested in vitro:

1. Under EBRT and HDR conditions, the linear attenuation coefficients (*μ*) of 3D-printed and heat-cured resin are equal.
2. The radiation attenuation of dental resins can be predicted satisfactorily as a linear function of thickness within the clinically relevant range of 6 to 14 mm.

## 2. Materials and Methods

**Figure 2:**
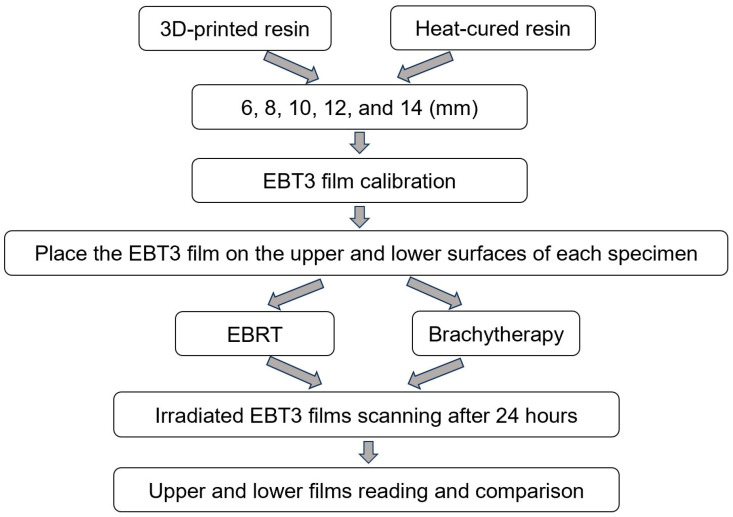
Flowchart of the experimental procedure.

A flowchart describing the experimental procedure is presented in Figure 2.

The study used two different types of resin. The first type is a photopolymer resin fabricated using 3D printing technology (Surgical guide; Formlabs Inc., Somerville, MA, USA). The second type is conventional heat-cured polymethyl methacrylate (Acron; GC Corp., Tokyo, Japan), which is widely used in dental and medical devices. Batch numbers and product information were taken from the company’s technical datasheet (Table 1).

### 2.1. Specimen Preparation

Specimens were 45 × 45 mm^2^ resins (heat-cured and 3D-printed) with five thicknesses (6, 8, 10, 12, and 14 mm), and ten specimens were made for each thickness of each material, for a total of 100 specimens. After preparation, all specimens were examined using a multi-slice CT scanner (Light Speed Xtra; GE Healthcare, Chicago, IL, USA) to confirm the thickness and lack of trapped air bubbles.

**Figure 3:**
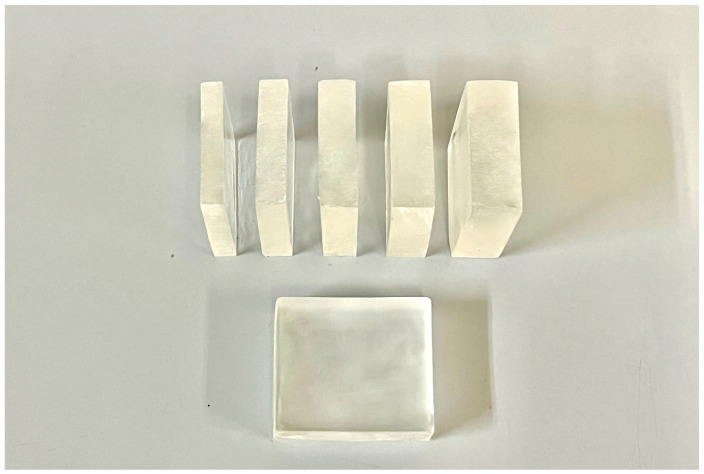
Heat-cured resin specimens with five different thicknesses.

Before fabricating the heat-cured resin specimens, molds with five different thicknesses were prepared. The heat-cured resin specimens were made following the manufacturer’s instructions with a powder/liquid ratio of 10 g/4 mL. The mixture is allowed to reach a doughy stage (about 15–20 min of setting time, depending on the material) before it is packed into the mold cavity and placed in a hot water polymerization unit. The recommended water temperature is maintained at 70–74 °C, with the curing process typically taking about 1.5 h at this temperature. Then, a final higher temperature cure (at around 100 °C) for 30 min was used to ensure complete polymerization. The polymerized specimens were removed from the molds and wet ground with successively finer grades of silicon carbide papers of from 500 to 1200 grit size to the predetermined dimensions (Figure 3).

**Figure 4:**
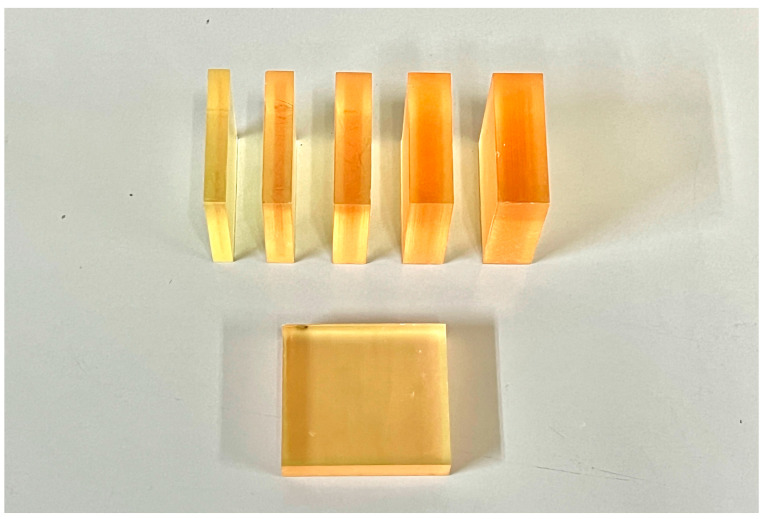
3D-printed resin specimens with five different thicknesses.

To fabricate 3D-printed resin specimens, open-source CAD software (Autodesk Fusion 360; Autodesk Inc., San Rafael, CA, USA) was used to design 45 × 45 mm^2^ specimens with thicknesses of 6, 8, 10, 12, or 14 mm. The final design was exported in an STL file, which was used to manufacture the specimens of the materials. The STL file was exported to a 3D printer slicer software program (Preform Software; Formlabs Inc., Somerville, MA, USA, Version 3.40.1). The liquid material was poured into the tank of a stereolithography 3D printer (Form 3B+; Formlabs Inc.). In the 3D printing process, the printer was configured for gapless printing to ensure the fabrication of homogeneous structures. The specimens were prepared for printing using the manufacturer’s parameters for exposure time, 50 µm layer thickness, and 90° printing orientation to the building platform surface. The printed parts were removed from the build platform and Form Wash (Formlabs Inc., Somerville, MA, USA) was used to remove any uncured resin. The specimens were then post-cured using Form Cure (Formlabs Inc., Somerville, MA, USA) to further harden the resin and achieve the desired material properties. The curing process was performed at 60 °C for 30 min. Then any support structures were carefully removed using the recommended tools and the surfaces were lightly sanded if necessary to smooth any remaining support marks. The specimens were inspected for any defects or irregularities before proceeding with further analysis (Figure 4).

### 2.2. Calibration of Radiochromic Film

For EBRT, radiochromic film (Gafchromic EBT3 film; Ashland Inc., Wilmington, DE, USA; batch number: 08032101) was exposed to a 6 MV photon beam within 12 radiation windows (4 × 6 cm^2^ each) of a linear accelerator (ClinaciX; Varian Medical Systems, Palo Alto, CA, USA), delivering doses ranging from 26.87 to 274.43 cGy. These absolute doses were determined according to the Japan Society of Medical Physics standard dosimetry 12 protocol [30].

For HDR, the EBT3 film (batch number: 08172201) was cut into 5 × 5 cm^2^ sized pieces and exposed to radiation from the HDR Ir-192 source (Flexitron system, Isodose Control, Veenendaal, Netherlands) with doses in the range of 50–1000 cGy. These absolute doses were determined according to the American Association of Physicists in Medicine AAPM TG-43 protocol [31].

The films were scanned 24 h after EBRT and HDR irradiation with a scanner (DS-G20000; Epson, Suwa, Japan) in RGB analysis mode with the red color channel, and the resulting image file was saved in JPEG format [31]. Using film dose analysis software (DD-analysis Ver. 10.10; R-Tech Inc., Tokyo, Japan), average pixel values (PV) were extracted from the scanned joint photographic experts group images. Calibration curves for EBRT and HDR were then created by correlating the extracted PV with the corresponding delivered radiation doses. Prior to irradiation, the unirradiated film was scanned as the background PV and the corresponding dose was set at 0 cGy. Subsequently, unknown doses could be measured by using calibration curves.

### 2.3. Phantom Assembly

**Figure 5:**
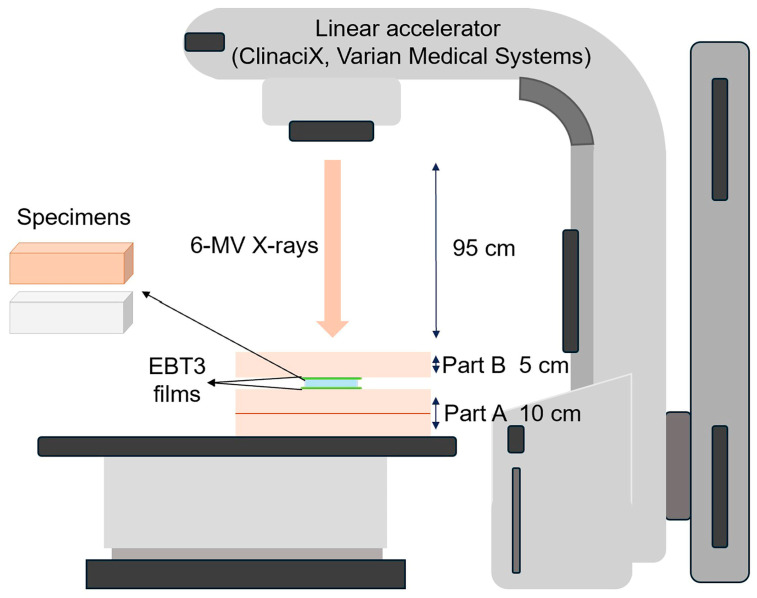
Schematic of the EBRT setup for exposing radiochromic film.

For EBRT, a water equivalent phantom (Tough water WE211; Kyoto Kagaku Co., Ltd., Kyoto, Japan) was used. Two pieces of radiochromic film were positioned horizontally above and below each specimen source. The specimen/film phantom assembly was placed on two tough water phantoms (part A), and then covered with an additional tough water phantom (part B) to provide full backscatter conditions. Figure 5 shows the setup of the source and EBT3 films in the phantom.

**Figure 6:**
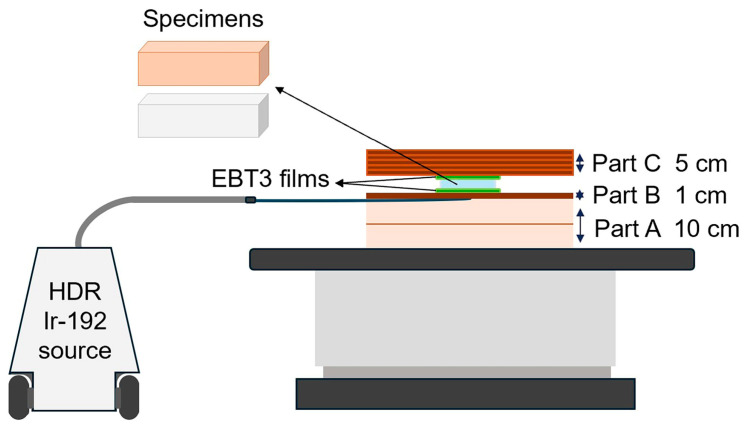
Schematic of the HDR setup for exposing radiochromic film.

For HDR, a water equivalent phantom and a tissue equivalent phantom (Bolus with skin; CIVCO Radiotherapy Inc., IA, USA) were used. The HDR Ir-192 source was fixed with tape on the water equivalent phantom (part A) and covered with the tissue equivalent phantom to provide a flat surface (part B). Two pieces of EBT3 film were positioned horizontally above and below each specimen source. The source/film part assembly was then covered with additional 30 × 30 × 5 cm^3^ tissue equivalent phantoms (part C) to provide full backscatter conditions. Arrangements of the source and EBT3 films in the phantom are shown in Figure 6.

### 2.4. Computational Method for EBT3 Film Measurement

The response of the EBT3 film to irradiation was expressed as the change in its PV. In image processing, we converted the PV to dose data using film dosimetry software (DD IMRT, version 12.42; R-Tec Inc., Tokyo, Japan) based on the calibration curves generated.

**Figure 7:**
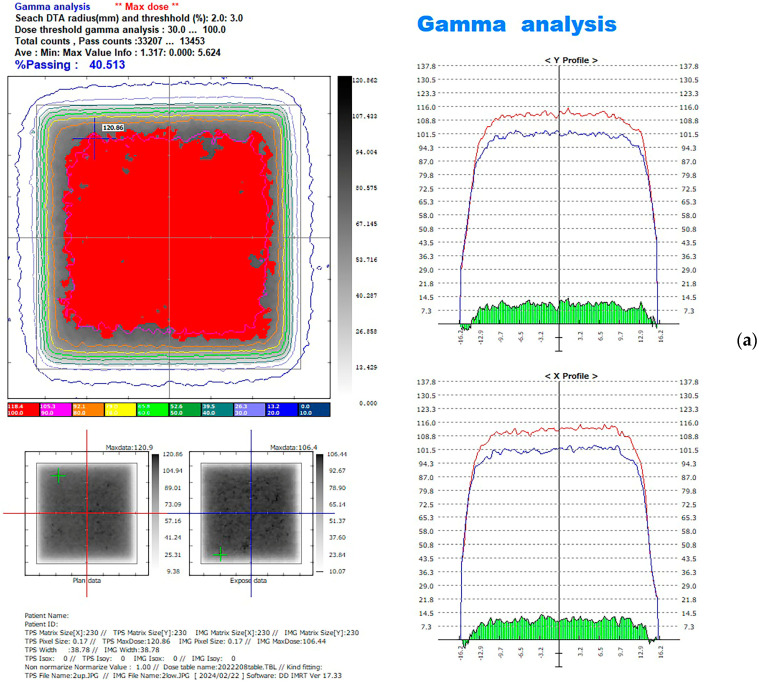

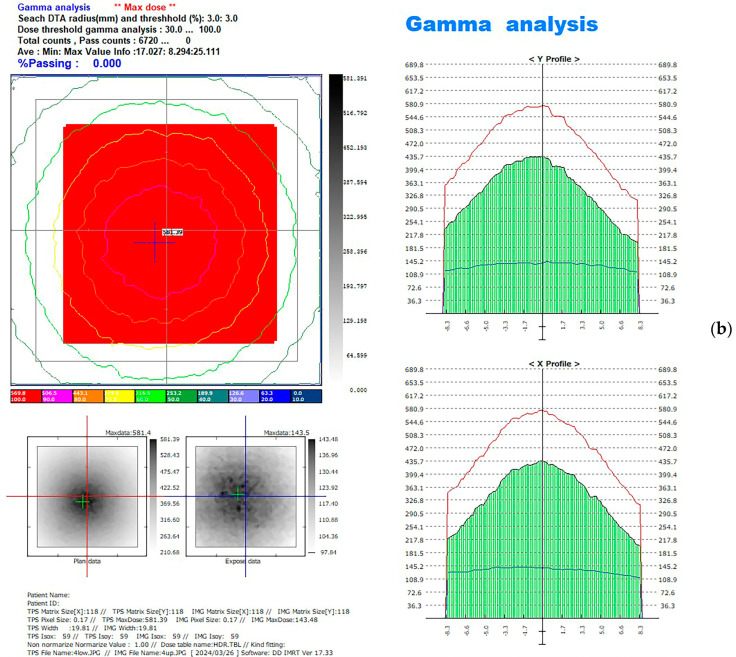
Doses of radiation from the upper and lower films on the X- and Y-axes. (**a**) EBRT; (**b**) HDR.

For EBRT, the desired doses for the 3 × 3 cm^2^ field of 150 MU using 6 MV X-rays were delivered by the linear accelerator. For HDR, 400 cGy was delivered by the Ir-192 source. After 24 h of irradiation, the films were scanned. The upper and lower films of the same specimen were compared by using the comparison function of the film dosimetry software, the red line represents the measured input radiation, and the blue line means the measured output radiation. (Figure 7). The data were exported into spreadsheets (Microsoft Excel; Microsoft Corp., Redmond, WA, USA, Version 2048). The doses for each film were added to the spreadsheet at a 0.353 mm pixel scale on both the X- and Y-axes. The doses in the 2 × 2 cm^2^ in the middle of each film were selected because the exposure was mostly uniform, whereas the doses of the margin on each side were excluded [32].

The attenuation of radiation intensity *I* within a material follows an exponential decay law, expressed by the following equation (*I* represents the radiation intensity after traversing the material. *I*_0_ denotes the initial radiation intensity before encountering the material. *μ* is the linear attenuation coefficient, a measure of how easily the material absorbs or scatters the radiation. Δ*x* indicates the thickness of the material through which the radiation passes):

*I* = *I_0_* exp(*−μ*Δ*x*).

The percentage radiation dose attenuation was calculated as follows:

For EBRT

Radiation attenuation (%) = [*mean dose measured on lower film* (*I*)/*mean dose measured on upper film* (*I*_0_)]∗100 (1)

For HDR

Radiation attenuation (%) = [*mean dose measured on upper film* (*I*)/*mean dose measured on lower film* (*I*_0_)]∗100 (2)

To transform the exponential attenuation relationship into a linear relationship, the natural logarithm of the above equation is taken:

ln(*I/I*_0_) = *−μ*Δ*x*

### 2.5. Statistical Analysis

All data were analyzed statistically using a statistical software program (IBM SPSS Statistics for Windows, version 28; IBM Corp., Armonk, NY, USA). The normality of the data distribution was determined by using the Shapiro–Wilk test, and the data homogeneity was confirmed by the Levene test. Independent two-tailed t-tests were performed to investigate differences in the radiation attenuation rates (*I*/*I*_0_) between the 3D-printed and heat-cured resins, with Bonferroni correction (α = 0.05). Linear regression models were obtained to analyze the relationships between the natural logarithm of the (*I*/*I*_0_***)*** and the Δ*x* of the resin materials, as well as between (*I*/*I*_0_) and the thickness (Δ*x*) of the resin materials. According to a post hoc power analysis conducted using G*Power software (version 3.1.9.2; Heinrich Hein University), the sample size of 50 samples for heat-cured resin (10 samples for each thickness) and 50 samples for 3D-printed resin (10 samples for each thickness) provided adequate statistical power to test the hypotheses in this study.

## 3. Results

### 3.1. Radiation Attenuation Comparison for EBRT

**Figure 8:**
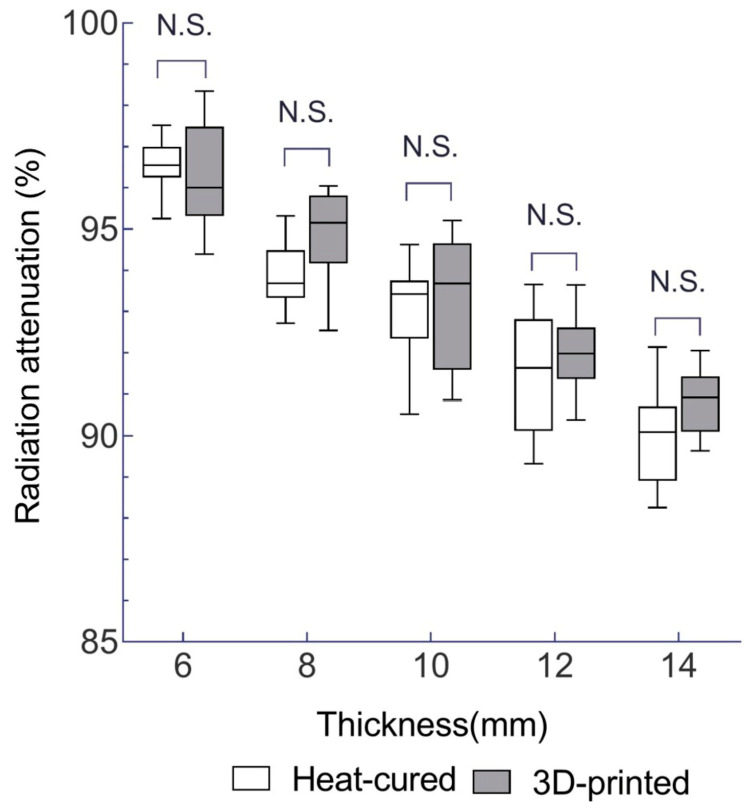
Mean radiation attenuation for 3D-printed and heat-cured resin after EBRT irradiation. N.S., no statistically significant difference (independent *t*-test followed by Bonferroni correction; adjusted *p* > 0.05).

According to the results of the independent t test, for EBRT, the differences in the mean radiation attenuation rate between the 3D-printed resin and heat-cured resin at each thickness of 6, 8, 10, 12, and 14 mm for EBRT were 0.2% (*p* = 1.000), 1% (*p* = 0.189), 0.3% (*p* = 1.000), 0.5% (*p* = 1.000), and 0.8% (*p* = 0.533), respectively. None of the differences were significant (*p*-values were Bonferroni corrected) (see Table 2 and Figure 8).

### 3.2. Radiation Attenuation Comparison for HDR

**Figure 9:**
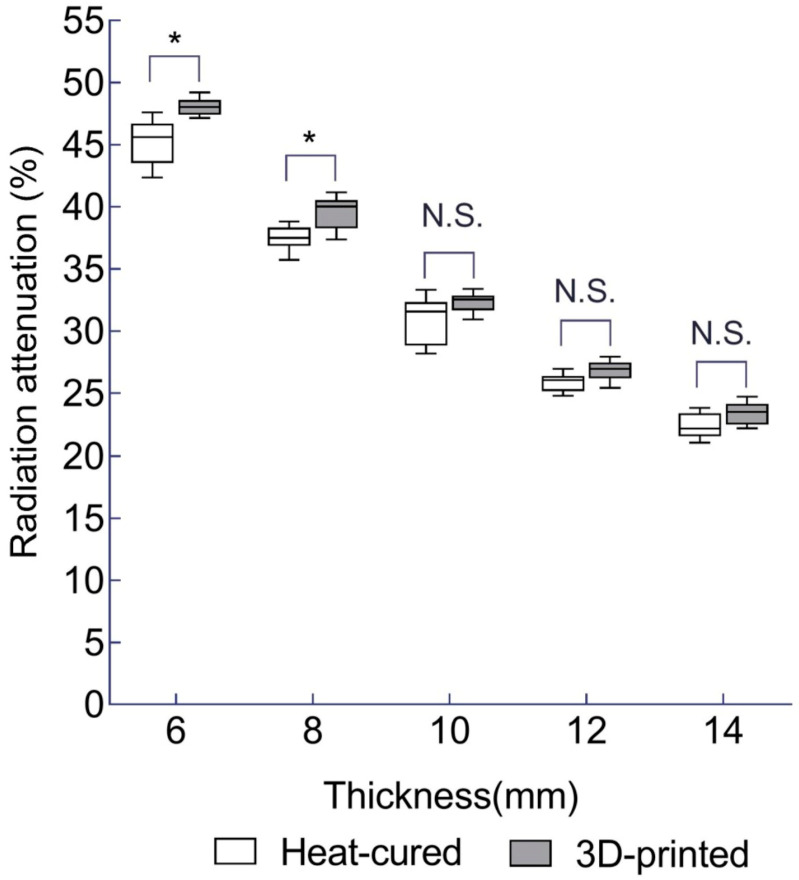
Mean radiation attenuation for 3D-printed and heat-cured resin after HDR irradiation. *—statistically significant difference (independent *t*-test followed by Bonferroni correction; adjusted *p* < 0.05). N.S.—no statistically significant difference.

For HDR, the differences in the mean radiation attenuation rate between the 3D-printed resin and heat-cured resin at each thickness of 6, 8, 10, 12, and 14 mm was 2.8% (*p* < 0.001), 2.1% (*p* = 0.005), 1.4% (*p* = 0.22), 0.9% (*p* = 0.08), and 1.0% (*p* = 0.135), respectively. Significant differences were observed at the 6 mm and 8 mm thicknesses (*p*-values were Bonferroni-corrected) (Table 3 and Figure 9).

### 3.3. Linear Regression between the ln (I/I_0_) and Thickness (Δx)

**Figure 10:**
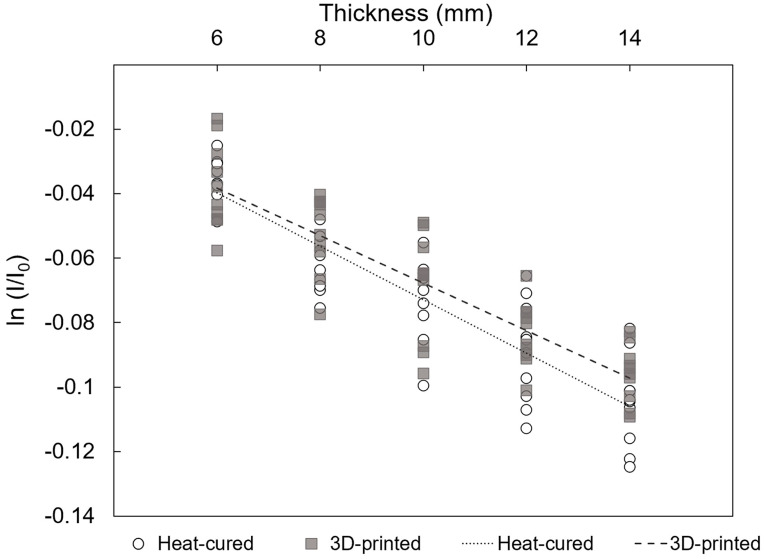
Linear regressions of natural logarithm of the attenuation rate versus thickness of resin materials under EBRT conditions (*p* < 0.001; adjusted r^2 ^= 0.789 and 0.750 for heat-cured and 3D-printed resin, respectively).

Linear regression analysis for EBRT revealed that the slope of the regression line relating the natural logarithm of the attenuation rate (ln (*I/I*_0_)) to the thickness (Δ*x*) was -0.0083 (95%CI; −0.0095 to −0.0070) for the heat-cured resin and −0.0074 (95%CI: −0.0086 to −0.0062) for the 3D-printed resin (*p* < 0.001) (Figure 10). These slopes represent the negative of the linear attenuation coefficient (*μ*), indicating attenuation coefficients of *μ* = 0.0083 mm^−1^ for the heat-cured resin and *μ* = 0.0074 mm^−1^ for the 3D-printed resin.

**Figure 11:**
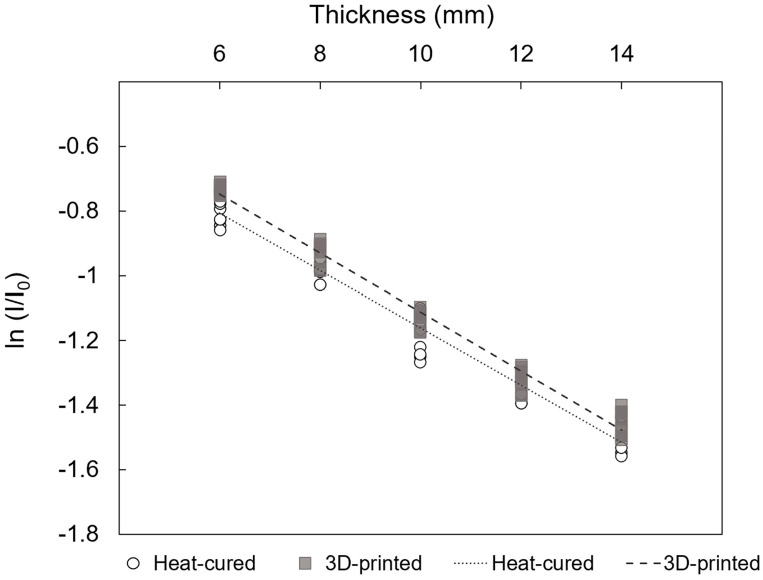
Linear regressions of natural logarithm of the attenuation rate versus thickness of resin materials under HDR conditions (*p* < 0.001; adjusted r^2^ = 0.971 and 0.983 for heat-cured and 3D-printed resin, respectively).

Figure 11 presents the linear regression analysis results for HDR, showing a slope of −0.0887 (95%CI: −0.0931 to −0.0843) for the heat-cured resin and −0.0914 (95%CI: −0.0948 to −0.0880) for the 3D-printed resin (*p* < 0.001), indicating attenuation coefficients of *μ* = 0.0887 mm^−1^ for the heat-cured resin and *μ* = 0.0914 mm^−1^ for the 3D-printed resin.

### 3.4. Linear Regression between the (I/I_0_) and Thickness (Δx)

**Figure 12:**
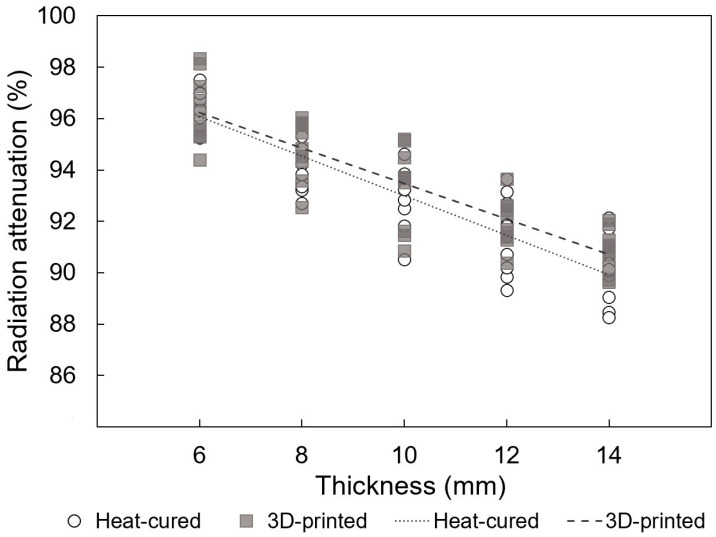
Linear regressions of radiation attenuation versus thickness of resin materials under EBRT conditions (*p* < 0.001; adjusted r^2^ = 0.749 and 0.791 for 3D-printed and heat-cured resin, respectively).

According to the linear regression analysis, for EBRT, the relationship is depicted in Figure 12, where a significant positive linear correlation between radiation attenuation rate and thickness is observed for both 3D-printed and heat-cured resin (*p* < 0.001; adjusted r^2^ = 0.749 and 0.791, respectively).

**Figure 13:**
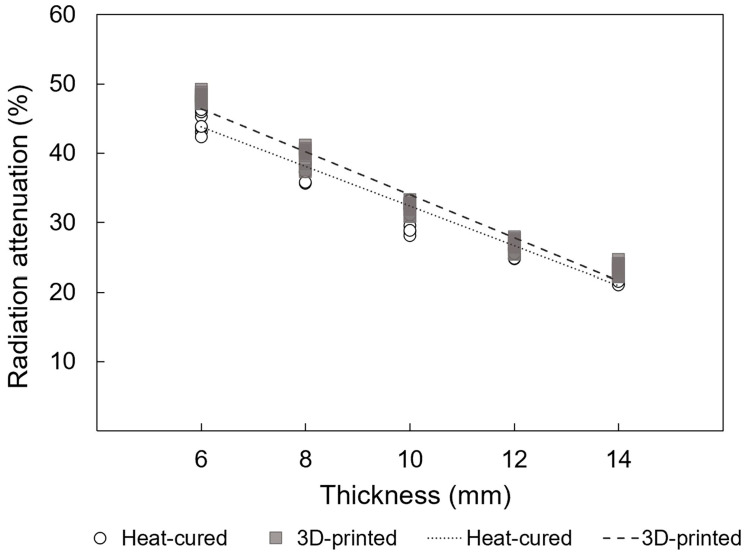
Linear regressions of radiation attenuation versus thickness of resin materials under HDR conditions (*p* < 0.001; adjusted r^2^ = 0.964 and 0.953 for 3D-printed and heat-cured resin, respectively).

Similarly, for HDR, Figure 13 illustrates strong positive linear correlations between radiation attenuation rates and thickness for both 3D-printed and heat-cured resin (*p* < 0.001; adjusted r^2^ = 0.964 and 0.953, respectively).

## 4. Discussion

Biocompatible 3D-printed resins are increasingly being explored beyond dental restorations and personalized orthodontic and prosthetic devices, extending to applications in radiotherapy devices for the head and neck region [6,7]. These devices aim to protect tissues outside the target area during radiation treatments, thereby reducing the side effects of radiotherapy [15,16,17,18]. To evaluate their potential use in radiotherapy prostheses, five different thicknesses of 3D-printed resin were tested in an in vitro simulation of EBRT and HDR treatments. The results were compared with those of traditional heat-cured resin, which has been widely used to date. Also, linear regression analysis was conducted to derive and compare the attenuation coefficients of 3D-printed and heat-cured resins under EBRT and HDR conditions. Given that the thickness of radiation prostheses can be tailored to individual patient needs, linear regression analysis was employed to assess the relationship between radiation attenuation and thickness within a clinically relevant range. The tested thickness range (6–14 mm) was chosen based on the effectiveness of radiotherapy prostheses in clinical settings and the tolerance of oral foreign bodies [17,18].

The impact of thickness on dose attenuation has been previously demonstrated in prior studies [26,29]. From the results of this study, no significant differences were found between the 3D-printed and heat-cured resins in EBRT, and significant differences were observed in HDR for the 6 and 8 mm thick specimens. Previous research has recommended that under HDR conditions, a radiotherapy prosthesis should be 10 mm thick to achieve a reduction in radiation intensity of 50% and minimize complications related to irradiation of the surrounding tissues [17,18]. In this in vitro experiment, the average reduction in radiation intensity rate of both the 3D-printed and heat-cured resins reached that standard (under HDR, at 6 mm thickness, 52.0% and 54.8% radiation intensity reduction for 3D-printed and heat-cured resins, respectively) and both 10 mm thick 3D-printed resin (67.7%) and heat-cured resin (69.1%) far surpassed the 50% threshold [29]. Additionally, linear regression analysis was used to calculate the linear attenuation coefficients. Under EBRT, the linear attenuation coefficient of the heat-cured resin was greater than that of the 3D-printed resin, while under HDR, the 3D-printed resin exhibited a higher attenuation coefficient. However, in both EBRT and HDR, the 95% confidence intervals for the attenuation coefficients of 3D-printed resin and heat-cured resin overlapped. These findings indicate that 3D-printed resin can provide sufficient radiation protection and comply with relevant standards and requirements.

Taniguchi et al., along with Obinata et al. and Fujita et al., recommended using radiotherapy prostheses that are as thick as possible to achieve the best protective effect [17,18,29]. However, in practical clinical applications, the customization of radiation prostheses should align with individual patient needs. For example, patients with limited mouth opening may require thinner prostheses, while those undergoing HDR treatment can tolerate thicker radiotherapy prostheses due to the shorter treatment duration. Experimental results indicate a linear relationship between radiation attenuation and thickness within the clinically relevant range, allowing for a rough estimation of how changes in prosthesis thickness might impact treatment outcomes.

Radiation attenuation also depends on the material’s physical qualities, particularly the atomic number and density [7]. Although there may be minor differences in the practical preparation processes because of various additives and stabilizers, the density of heat-cured resin (1.20 g/cm^3^) is higher than that of 3D-printed resin (1.19 g/cm^3^), which may contribute to a slight edge observed in the performance of the heat-cured group compared to the 3D-printed group.

At the same time, the radiation source is also one of the factors affecting material attenuation [27,28]. Based on the observed results, it is evident that both the 3D-printed and heat-cured specimens of the same thickness exhibited notably lower radiation attenuation under the EBRT compared to the HDR source. Specifically, the radiation reduction ranged from 3.7% to 9.1% for 3D-printed resin and from 3.4% to 10.0% for heat-cured resin under EBRT. The differences in performance of the same material under EBRT and HDR mainly stem from the fact that, although both treatments use photon beams, EBRT employs 6 MV X-rays, while HDR uses an Ir-192 source with an average γ-ray energy of 380 keV. While distance can also impact radiation intensity, in our experimental setup, doses were measured before the beam reached the material. Therefore, variations in distance and solid angle did not affect our results. The differences in attenuation observed in our experiment are primarily attributed to the differences in radiation source energy, rather than the relative position or distance of the sample

In this study, 3D-printed resin showed promising radiation protection characteristics, making it a promising new choice for constructing radiotherapy prostheses. Unlike the traditional process of making radiotherapy prostheses, which typically take around 2 weeks, 3D-printed radiotherapy prostheses can be completed in just a few hours [13]. This indicates that 3D-printed resin has advantages as an alternative in radiation applications. Additionally, the limited references on this specific topic highlight the novelty and originality of this research.

The first limitation of this study is the utilization of only one type of EBRT and brachytherapy source. Although the experimental outcomes may be extrapolated to predict performance under various radiation modalities, future investigations should explore material performance under different types of radiation and radiation energy levels to ensure more robust results. The second limitation pertains to the selection of only one representative 3D material for this experiment. It is acknowledged that further experiments are necessary to investigate other 3D materials that have become available in recent years. Additionally, this study is an in vitro design, which cannot perfectly simulate clinical conditions. Therefore, these limitations prompt a desire to extend the research to other potential resins.

## 5. Conclusions

Based on the findings of this in vitro study, the following conclusions were drawn:

1. The 3D-printed resin exhibits slightly different radiation attenuation coefficients compared to heat-cured PMMA, and the 95% confidence intervals overlapped. This suggests that 3D-printed resin can be considered a viable alternative for radiotherapy prostheses.
2. Within a clinically relevant thickness range (6–14 mm), the radiation attenuation rate shows a strong linear relationship with resin thickness. This indicates that linear estimation can be roughly used to predict radiation shielding effectiveness based on thickness.

## Figures and Tables

**Table 1:** Summary of resin materials investigated in this study.

Brand Name	Manufacturer	Processing Technique	Resin Composition	Lot	Density (g/cm^3^)
ACRON	GC Corp.	Heat polymerization	Powder: methacrylic acid ester polymer Liquid: methyl methacrylate	Powder: 2206021 Liquid: 2205061	1.20
Surgical guide resin	Formlabs	Photopolymerization	Urethane dimethacrylate, methacrylate monomer (s), photoinitiator (s)	SG01220531-01	1.19

**Table 2:** Radiation attenuation average and standard deviation of the two resin materials under EBRT.

Thickness (mm)	Radiation Attenuation (%)	
	3D-Printed Resin	Heat-Cured Resin
6	96.3 ± 1.3	96.5 ± 0.6
8	94.9 ± 1.1	93.9 ± 0.8
10	93.3 ± 1.5	93.0 ± 1.2
12	92.0 ± 0.9	91.5 ± 1.4
14	90.8 ± 0.8	90.0 ± 1.2

**Table 3:** Radiation attenuation average and standard deviation of the two resin materials under HDR.

Thickness (mm)	Radiation Attenuation (%)	
	3D-Printed Resin	Heat-Cured Resin
6	48.0 ± 0.7	45.2 ± 1.9
8	39.6 ± 1.3	37.5 ± 1.1
10	32.3 ± 0.8	30.9 ± 1.9
12	26.8 ± 0.9	25.9 ± 0.7
14	23.4 ± 0.9	22.4 ± 1.0

## Data Availability

The original contributions presented in the study are included in the article, further inquiries can be directed to the corresponding author.
